# Availability of medical cannabis services by racial, social, and geographic characteristics of neighborhoods in New York: a cross-sectional study

**DOI:** 10.1186/s12889-022-13076-1

**Published:** 2022-04-06

**Authors:** Chinazo O. Cunningham, Chenshu Zhang, Maegan Hollins, Melinda Wang, Sumeet Singh-Tan, Paul J. Joudrey

**Affiliations:** 1 Albert Einstein College of Medicine, 1300 Morris Park Ave, Bronx, NY 10461 USA; 2 Northwestern University, 633 Clark St, Evanston, IL 60208 USA; 3 Yale School of Medicine, 333 Cedar St, New Haven, CT 06510 USA

**Keywords:** Medical cannabis, New York, Racial and ethnic disparities, Census tract, Availability of legal cannabis

## Abstract

**Background:**

Within the United States (US), because racial/ethnic disparities in cannabis arrests continue, and cannabis legalization is expanding, understanding disparities in availability of legal cannabis services is important. Few studies report mixed findings regarding disparities in availability of legal cannabis services; none examined New York. We examined disparities in availability of medical cannabis services in New York. We hypothesized that New York census tracts with few Black or Hispanic residents, high incomes, high education levels, and greater urbanicity would have more medical cannabis services.

**Methods:**

In this cross-sectional study, we used data from the 2018 US Census Bureau 5-year American Community Survey and New York Medical Marijuana Program. Main exposures were census tract characteristics, including urban–rural classification, percentage of Black and Hispanic residents, percentage of residents with bachelor’s degrees or higher, and median household income. Main outcomes were presence of at least one medical cannabis certifying provider and dispensary in each census tract. To compare census tracts’ characteristics with (vs. without) certifying providers and dispensaries, we used chi-square tests and t-tests. To examine characteristics independently associated with (vs. without) certifying providers, we used multivariable logistic regression.

**Results:**

Of 4858 New York census tracts, 1073 (22.1%) had medical cannabis certifying providers and 37 (0.8%) had dispensaries. Compared to urban census tracts, suburban census tracts were 62% less likely to have at least one certifying provider (aOR = 0.38; 95% CI = 0.25–0.57). For every 10% increase in the proportion of Black residents, a census tract was 5% less likely to have at least one certifying provider (aOR = 0.95; 95% CI = 0.92–0.99). For every 10% increase in the proportion of residents with bachelor’s degrees or higher, a census tract was 30% more likely to have at least one certifying provider (aOR = 1.30; 95% CI = 1.21–1.38). Census tracts with (vs. without) dispensaries were more likely to have a higher percentage of residents with bachelor’s degrees or higher (43.7% vs. 34.1%, *p* < 0.005).

**Conclusions:**

In New York, medical cannabis services are least available in neighborhoods with Black residents and most available in urban neighborhoods with highly educated residents. Benefits of legal cannabis must be shared by communities disproportionately harmed by illegal cannabis.

## Background

As advances in medical treatment emerge, the inequitable distribution of these medical advances among Americans can exacerbate health disparities [1]. Although cannabis has been used for medicinal purposes for centuries, in the United States (US), the legal use of medical cannabis is a relatively new phenomenon [2]. Currently, the District of Columbia and 17 states, including New York, have legalized adult-use recreational cannabis, and the District of Columbia and 36 states have legalized medical cannabis [3]. In states where recreational cannabis use remains criminalized, the benefits of access to medical cannabis, including availability (i.e. geographic location) and affordability (i.e. direct cost of the service for patients), may extend beyond medical benefits.

In the US, the harms of cannabis criminalization have disproportionately impacted Black and Hispanic people [4, 5]. Across the US and New York, the arrest rates for cannabis possession are 3.6 and 2.6 times higher for Black people than White people, respectively [4]. In several counties in New York, the arrest rates are over 15 times higher for Black people than White people [4]. Arrest rates for cannabis possession are similarly higher in communities with lower income and levels of education [6]. New York law did not distinguish between medical and recreational cannabis use until the enactment of medical cannabis. Because legal medical cannabis should allow individuals to use medical cannabis without risk of criminal sanction, access to medical cannabis certification within Black and Hispanic communities could reduce the harm of cannabis criminalization for these populations, particularly for those with an indication for medical cannabis who may otherwise obtain cannabis from the illicit market. Therefore, if medical cannabis is less available within Black and Hispanic communities, then the impact of medical cannabis policies on racial inequities may be reduced.

US federal and state policies related to cannabis differ substantially, impacting how people legally access cannabis. At the federal level, cannabis remains classified as a Schedule 1 substance with “no currently accepted medical use and a high potential for abuse” [7]. This classification, which includes substances like heroin, has important clinical implications. Because no insurance company covers medical cannabis products, patients must pay out of pocket with cash. In addition, medical providers cannot *prescribe* medical cannabis products, but instead *certify* patients for medical cannabis. Certification for medical cannabis by a medical provider is a necessary first step to obtaining medical cannabis products. In many states, including New York, a certified patient can access medical cannabis at a limited number of approved dispensaries.

To our knowledge, no previous study has examined whether and the degree to which disparities in availability of medical cannabis services exist in New York. We examined how neighborhood racial, social, and geographic characteristics differed by locations in which certifying providers and medical cannabis dispensaries were present in New York. Specifically, we compared racial and ethnic composition, income, education, and urbanicity of New York census tracts by presence of certifying providers and medical cannabis dispensaries. Informed by evidence of inequities by race, ethnicity, income, and geography for other medical innovations [1, 8], we hypothesized that New York census tracts with 1) few Black or Hispanic residents, 2) high incomes, 3) high education levels and 4) greater urbanicity would be most likely to have certifying providers and medical cannabis dispensaries.

## Methods

### Setting

The cross-sectional study was conducted out of the Albert Einstein College of Medicine (Bronx, New York), and its institutional review board approved this study (IRB# 2020–11913). All methods were carried out in accordance with relevant guidelines and regulations.

The New York Medical Marijuana Program was implemented in January 2017. As of June 16, 2020, 116,518 patients were enrolled in the program, and 2267 certifying providers and 38 medical cannabis dispensaries were listed on the program website [9]. To become a certifying provider, physicians, nurse practitioners, or physician assistants must complete a 4-h online educational course on cannabinoids and register with the New York State Department of Health. Within the program, 10 registered organizations are responsible for manufacturing and dispensing medical cannabis products, and each is permitted to have up to four dispensaries. Although New York legalized adult-use recreational cannabis on March 31, 2021, implementation of the policy is not yet in effect.

### Study population

Of the 4918 census tracts in New York, 17 had a population of zero and 43 were missing rural–urban data codes. Therefore, we excluded these 60 census tracts and included a total of 4858 census tracts in analyses.

### Study variables

#### Dependent variables

The main dependent variable was the presence of at least one certifying provider’s address in each census tract (yes/no). Because the majority of census tracts had no certifying provider, we defined this variable based on the presence of any certifying provider in each census tract instead of the number of certifying providers in each census tract. To create this variable, we first abstracted names and addresses of all 2267 certifying providers who were listed on the New York Medical Marijuana Program website as of June 16, 2020. These 2267 certifying providers had 2310 addresses; 2225 providers had one address, 41 had two addresses, and 1 had three addresses. Forty-three providers had addresses only outside of New York, and 2224 had at least one address in New York. If applicable, we included certifying providers with multiple addresses in multiple census tracts.

We then obtained the legal boundaries of New York census tracts from the 2019 US Census Bureau TIGER/Line Shapefiles [10]. To geocode and map certifying providers’ addresses, we used Esri ArcPro 2.2 and ArcGIS online geocoding service to complete a process that we have employed previously in accordance with best practices [11–14]. During batch geocoding (assigning latitude and longitude to a database of addresses), 2262 of 2310 address were successfully matched to a location. The remaining 48 addresses were initially matched during batch geocoding to a polygon location (i.e. center point of postal code or municipality), had a tied highest match score, or had a match score of less than 80. All 48 of these addresses were then successfully matched during hand review using Google Maps. Of the 2310 addresses, 44 were outside of New York. Thus, we mapped the 2266 unique New York geocoded addresses of certifying providers over the New York census tract boundaries to assign each certifying provider’s address to a specific census tract.

Our other dependent variable was the presence of at least one medical cannabis dispensary in each census tract (yes/no). We used a similar approach as described above, but used addresses of the 38 medical cannabis dispensaries (rather than certifying providers) obtained from the New York Medical Marijuana Program website. During batch geocoding, 37 of 38 dispensaries’ addresses were successfully located, and one address was successfully located upon hand review.

#### Independent variables

Key independent variables included urban–rural classification, racial and ethnic composition, and education and income levels for each census tract.

Using the 2019 revised Rural–Urban Commuting Area (RUCA) codes from the US Department of Agriculture [15], we categorized census tracts into a commonly used 3-level urban–rural classification scheme [16, 17]: urban (RUCA codes 1.0, 1.1), suburban (RUCA codes 2.0, 2.1, 3.0), and rural (RUCA codes 4.0, 4.1, 4.2, 5.0, 5.1,5.2,6.0, 6.1, 7.0, 7.1, 7.2, 7.3, 7.4,8.0, 8.1, 8.2, 8.3, 8.4, 9.0,9.1, 9.2,10.0, 10.1, 10.2, 10.3, 10.4, 10.5, 10.6). Because our study did not aim to examine difference across rural communities, we collapsed the previously used 4-level scheme (urban, suburban, large rural, and small rural) into a 3-level scheme by combining large rural and small rural categories.

Using the 2018 US Census Bureau 5-year American Community Survey [18], we obtained demographic, educational and economic data for each census tract. For racial and ethnic composition, we calculated the mean percentage of Black residents (including Hispanic and non-Hispanic ethnicity), Hispanic residents (including all races). For education, we calculated the mean percentage of residents 25 years of age or older with bachelor’s degrees or higher. For income levels, we calculated the mean median household income in 2018 inflation-adjusted dollars. All variables and categories were based on those defined in the 2018 US Census Bureau 5-year American Community Survey.

#### Other variables

To provide descriptive data on certifying provider characteristics from the publicly available New York State Office of Professions website [19], we collected provider type (Medical Doctor [MD], Doctor of Osteopathy [DO], Nurse Practitioner [NP], Physician Assistant [PA]), and date of medical degree (available for MDs and DOs).

### Data analyses

The unit of analysis was census tract. To compare characteristics of census tracts with versus without at least one certifying provider, we conducted bivariate analyses using chi-square tests for categorical variables and t-tests for continuous variables. To examine whether characteristics of census tracts were independently associated with the presence of certifying providers, we then conducted a multivariable logistic regression model in which all independent variables were included in the model. Due to missing values for independent variables, 164 observations (3.4%) were excluded from the model. Because the categories of urban–rural classification take population density into account, we did not include population density in our model. We chose urban census tracts as the reference group because it is the most frequent urban–rural classification of census tracts. We performed a Hosmer and Lemeshow goodness-of-fit test to examine model fit of the data. We also conducted a sensitivity analysis to examine if spatial autocorrelation impacted our findings. We tested for spatial autocorrelation for our main dependent variable using Moran's I. We then repeated our multivariable logistic regression by incorporating spatial autocorrelation mixed-effect structure into the model.

To compare characteristics of census tracts with versus without at least one medical cannabis dispensary, we conducted bivariate analyses using chi-square tests for categorical variables and t-tests for continuous variables. Because only 38 medical cannabis dispensaries were present in 37 of New York’s census tracts, we did not conduct multivariable analyses.

Finally, to describe characteristics of certifying providers, we ran simple frequencies of their characteristics, combining variables into clinically meaningful categories. Analyses were conducted using SAS 9.4 (Cary, NC, USA).

## Results

**Fig. 1:**
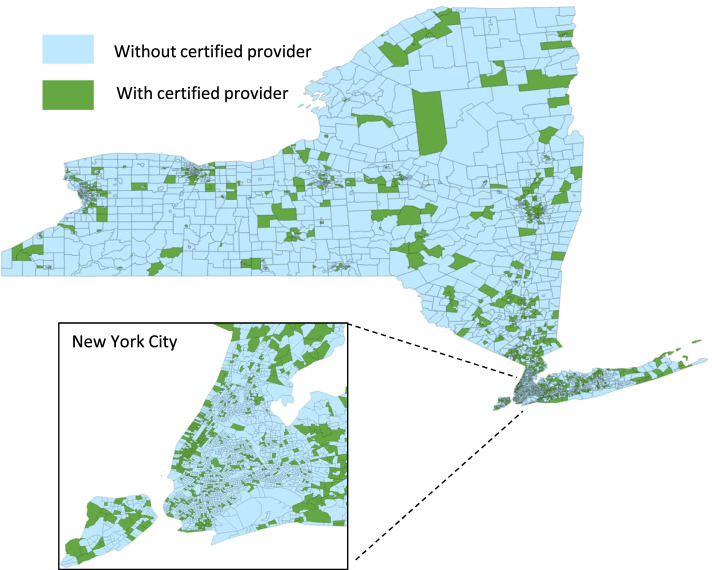
New York census tracts with and without medical cannabis certifying providers

**Fig. 2:**
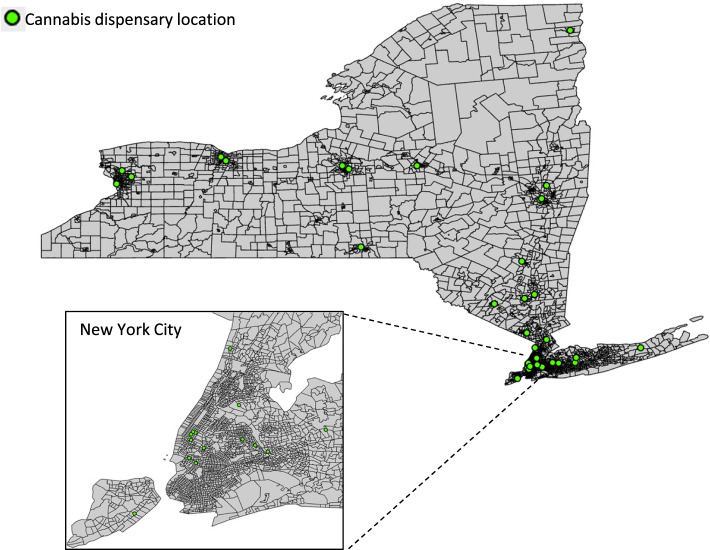
New York census tracts with and without medical cannabis dispensaries

Of the 4858 New York census tracts, 1073 (22.1%) had at least one certifying provider and 37 (0.8%) had at least one medical cannabis dispensary (Table 1, Figs. 1 and 2). Of 2224 certifying providers with New York addresses, most were physicians (60.4% MDs, 6.7% DOs) or nurse practitioners (29.5%). A majority (63.7%) of physicians received medical degrees over 20 years ago. Certifying providers’ most common specialties were family medicine (20.8%), general internal medicine (19.1%), psychiatry (12.3%), pain medicine (10.8%), neurology (8.1%), anesthesiology (4.7%), and physical medicine and rehabilitation (4.8%).

**Table 1:** New York census tract characteristics by presence of medical cannabis certifying providers and dispensaries

Census tract characteristic	All census tracts	Census tracts by certifying providers			Census tracts by medical cannabis dispensaries		
	(*N* = 4858)	With certifying providers (*N* = 1073)	Without certifying providers (*N* = 3785)	p-value	With medical cannabis dispensaries (*N* = 37)	Without medical cannabis dispensaries (*N* = 4821)	p-value
Geographic characteristic, n (%)							
Urban	4065 (83.7)	957 (89.2)	3108 (82.1)	< 0.001	36 (97.3)	4029 (83.6)	0.08
Suburban	321 (6.6)	28 (2.6)	293 (7.7)		0 (0)	321 (6.7)	
Rural	472 (9.7)	88 (8.2)	384 (10.2)		1 (2.7)	471 (9.8)	
Percent of Hispanic residents, mean (SD)	17.8% (19.6%)	15.0% (16.3%)	18.6% (20.3%)	< 0.001	15.9% (13.7%)	17.8% (19.6%)	0.40
Percent of Black residents, mean (SD)	18.3% (25.1%)	13.6% (20.0%)	19.6% (26.2%)	< 0.001	13.3% (15.3%)	18.3% (25.1%)	0.06
Median household income in $USD, mean (SD)	$84,123 ($40,908)	$99,082 ($47,960)	$80,001 ($37,719)	< 0.001	$93,449 ($43,487)	$84,055 ($40,886)	0.18
Percent of residents with bachelor’s degree or higher, mean (SD)	34.1% (19.0%)	43.4% (21.2%)	31.5% (17.4%)	< 0.001	43.7% (22.7%)	34.1% (18.9%)	0.002

Missing data: Percent of Hispanic residents = 21; Percent of Black residents = 21; Median household income = 164; Percent of residents with bachelor’s degree or higher = 25

In bivariate analyses, census tracts with (vs. without) at least one certifying provider were less likely to be suburban (2.6% vs. 7.7%, *p* < 0.001), and more likely to be urban (89.2% vs. 82.1%, *p* < 0.001), have a lower percentage of Black and Hispanic residents (13.6% vs. 19.6%, *p* < 0.001; 15.0% vs. 18.6%, *p* < 0.001, respectively), have a higher median household income ($99,082 vs. $80,001, *p* < 0.001), and have a higher percentage of residents with bachelor’s degrees or higher (43.4% vs. 31.5%, *p* < 0.001) (Table 1). Census tracts with (vs. without) at least one medical cannabis dispensary were more likely to have a higher percentage of residents with bachelor’s degrees or higher (43.7% vs. 34.1%, *p* = 0.002).

In multivariable analysis, compared to urban census tracts, suburban census tracts were 62% less likely to have at least one certifying provider (aOR = 0.38; 95% CI = 0.25–0.57) while there was no difference detected between urban and rural census tracts. For every 10% increase in the proportion of Black residents, a census tract was 5% less likely to have at least one certifying provider (aOR = 0.95; 95% CI = 0.92–0.99). For every 10% increase in the proportion of residents with bachelor’s degrees or higher, a census tract was 30% more likely to have at least one certifying provider (aOR = 1.30; 95% CI = 1.21–1.38, Table 2). For this model, the Hosmer and Lemeshow chi-square was 10.21 (*p* = 0.25), the Cox and Snell R-square was 0.06, and the Nagelker R-square was 0.09. In our sensitivity analysis, there was evidence of spatial autocorrelation for our primary outcome (Moran’s Index 0.03; *p* < 0.001). However, our model with a mixed-effect term for nearby census tracts with medical cannabis certifiers did not impact our primary findings (data not shown).

**Table 2:** Multivariable logistic regression model examining characteristics of New York census tracts with versus without medical cannabis certifying providers (*N* = 4694)

Census tract characteristic	aOR (95% CI)	p-value
Geographic characteristic		
Urban	ref	
Suburban	0.38 (0.25–0.57)	< 0.001
Rural	1.01 (0.77–1.33)	0.94
Percent of Hispanic residents (per 10% increase)	0.98 (0.94–1.03)	0.44
Percent of Black residents (per 10% increase)	0.95 (0.92–0.99)	0.007
Median household income (per $10,000 USD increase)	1.00 (0.98–1.03)	0.89
Percent of residents with bachelor’s degree or higher (per 10% increase)	1.30 (1.21–1.38)	< 0.001

Model statistics:

Likelihood Ratio Chi-square for Global Null Hypothesis: Beta = 0, Chi-square = 276.5, DF = 6, p-value < 0.001

-2 Log Likelihood value = 4623

Hosmer and Lemeshow Goodness-of-Fit Test: Chi-square = 10.2, DF = 8, p-value = 0.25

Nagelker R-square = 0.09

Cox and Snell R-square = 0.06

## Discussion

In the New York Medical Marijuana Program, medical providers who certify individuals for medical cannabis are located in less than one quarter of census tracts, and dispensaries that provide medical cannabis products are located in less than 1% of census tracts. Certifying providers are most likely to be in census tracts with fewer Black residents (hypothesis #1) and more highly educated residents (hypothesis #3). Certifying providers are more likely to be in urban versus suburban census tracts, but we did not find evidence of a difference in availability between urban and rural census tracts (hypothesis #4). We did not find evidence that certifying provider availability was associated with census tract income (hypothesis #2). Medical cannabis dispensaries may be located in similar locations, though due to the small number of dispensaries, independent associations could not be determined.

Our finding of racial and educational disparities in availability of medical cannabis services in New York is not surprising. Findings similar to ours, in which Black patients have less access than white patients to advances in health care, have been documented repeatedly and across numerous medical conditions. Examples of well-documented racial disparities in access to advances in health care include: buprenorphine treatment for opioid use disorder [20, 21], highly active antiretroviral medication for HIV infection [22–24], joint replacements for arthritis [25, 26], interventions for acute myocardial infarction [27, 28], and renal transplant for end stage renal disease [29, 30]. Similarly, low educational levels have been previously associated with less access to medical, dental, and mental health care [31]. However, our finding that neighborhoods with a greater proportion of Black New Yorkers were less likely to have available medical cannabis services is particularly problematic given the historical and ongoing disproportionate rates of cannabis arrests among Black people relative to white people in New York and the US [4]. As more states legalize recreational and medical cannabis, policies that do not take into account racial disparities in the harms of cannabis criminalization may perpetuate racial disparities. Additional interventions or reparations targeted to the most harmed communities is likely to be required to reduce racial disparities associated with cannabis use in the US.

To our knowledge, our study is among the first to examine how location of certifying providers is associated with sociodemographic characteristics of the population they serve. In addition, our study is the first to examine medical cannabis services in New York, which has one of the most stringent medical cannabis policies in the US. In the only study that we are aware of that examined disparities in medical cannabis certification, only 14% of patients assessed for certification at nine medical cannabis clinics in California were Hispanic, while 32% of Californians were Hispanic [32]. Our study did not find a relationship between the percentage of Hispanic residents in a census tract and the availability of cannabis services. Future research is needed to confirm this negative finding and also examine how neighborhood availability of services is related to certification rates. Other studies have focused on geospatial locations of medical or recreational cannabis dispensaries, not certifying providers. While most of these studies report that dispensaries were more commonly located in neighborhoods with high levels of poverty or deprivation, or high percentages of Black or Hispanic residents [33–37], one study reported no relationship between locations of dispensaries and these neighborhood characteristics [38]. There are several potential reasons for the differences in findings between these studies and ours. Other studies often combined medical cannabis and recreational cannabis dispensaries, while New York has only medical cannabis dispensaries. In addition, New York has only 38 medical cannabis dispensaries, while California, Washington, and Colorado have hundreds of dispensaries [36, 39, 40]; thus, the limited number of dispensaries in New York may be strategically located in select neighborhoods. In our clinical experience, the cost of medical cannabis is New York is often unaffordable to many, which is also likely to contribute to decisions regarding locations of dispensaries. Although 36 states have legalized medical cannabis, relatively few studies, and only studies from California, Washington, and Colorado, have examined how medical cannabis policies are associated with access to medical cannabis, particularly by race and ethnicity, along with income and education. Our results are consistent with prior research showing greater availability of medical services within urban versus suburban areas [8, 41]. However, our results differ from previous research that showed less availability in rural versus urban areas [8, 13]. Our results for rural areas was unexpected, but future research should examine disparities using other measures of availability, such as drive time to services. Understanding and mitigating disparities in access to advances in health care, and specifically legal cannabis, is critical to ensuring equitable health care delivery and outcomes.

The current New York Medical Marijuana program does not include measures to encourage the adoption of medical cannabis certification among providers within communities harmed by cannabis criminalization. For medical cannabis, our results suggest incentives to provide services within such communities are needed if all potential benefits of legal medical cannabis are to be realized. One potential option is to incentivize or encourage the adoption of medical cannabis certification among providers at Federally Qualified Health Centers, which disproportionately serve under-resourced communities. States like New York could also provide financial incentives to open new practices that provide medical cannabis certification within communities harmed by cannabis criminalization. Our results also suggest New York should increase the number of medical cannabis dispensaries and incentivize their location within communities harmed by cannabis criminalization to avoid disparities in dispensary availability. Despite the shortcomings in New York’s Medical Marijuana Program, the recently passed law for legal adult-use cannabis in New York has earmarked 40% of tax revenues to be invested in communities, along with social and economic equity programs to address those disproportionally impacted by cannabis criminalization [42]. These provisions include preferences for licensing to minority-owned businesses. It will be important to evaluate whether these provisions lead to equitable availability and access to legal cannabis.

Our study has limitations. Because New York’s medical cannabis policies differ from other states in the US, our findings may not be generalizable to other states. Because we used a dichotomous outcome for availability of services, we could not assess differences in the density of certifying providers among census tracts. New York also allows medical cannabis certification and dispensary visits via telemedicine, along with delivery of medical cannabis products. While telemedicine may potentially reduce the importance of geographic proximity, adoption and availability of telemedicine may align with the same racial disparities identified within our study [43]. Because Black race and Hispanic ethnicity are defined by the 2018 US Census Bureau 5-year American Community Survey, we were unable to combine or parse out these variables to isolate individuals who may have identified as Black and Hispanic. We did not include other measures, such as cost or stigma of medical cannabis use, which are likely to impact disparities in access to medical cannabis services and products. Because this was a cross-sectional study, future research should examine how these results may change over time. While the exact proportion of medical versus recreational cannabis users within New York is unknown, the approved conditions for medical cannabis within New York are present within all racial and ethnic subpopulations and these populations have an equal right to access medical cannabis free of criminal penalty [9]. Despite these limitations, we remain confident in our robust findings on the relationship between the availability of medical cannabis and the percentage of Black residents within a neighborhood.

## Conclusions

In the New York Medical Marijuana Program, certifying providers are less likely to be located in neighborhoods with high percentages of Black residents, and more likely to be located in neighborhoods that are urban and with highly educated residents. Given the historical and ongoing racial disparities in cannabis arrests across the US and New York, ensuring equitable access to legal cannabis services and products is critical and just. As legalization of cannabis continues to grow, states need to take additional measures to ensure equitable availability of and access to the benefits of cannabis legalization.

## Data Availability

The datasets analyzed in the study are publicly available as follows: 1) the New York State Medical Marijuana Program at https://www.health.ny.gov/regulations/medical_marijuana/, 2) New York State Office of Professions website at http://www.op.nysed.gov/opsearches.htm#nme, 3) 2018 US Census Bureau 5-year American Community Survey at https://www.census.gov/acs/www/data/data-tables-and-tools/data-profiles/2018/, and 4) 2019 US Census Bureau TIGER/Line Shapefiles at https://www.census.gov/geographies/mapping-files/time-series/geo/tiger-line-file.html. The datasets used and/or analyzed during the current study are available from the corresponding author upon reasonable request.
